# Radioimmunotheragnosis in Cancer Research

**DOI:** 10.3390/cancers16162896

**Published:** 2024-08-20

**Authors:** Guillermo Garaulet, Bárbara Beatriz Báez, Guillermo Medrano, María Rivas-Sánchez, David Sánchez-Alonso, Jorge L. Martinez-Torrecuadrada, Francisca Mulero

**Affiliations:** 1 Molecular Imaging Unit, Spanish National Cancer Center—CNIO, 28029 Madrid, Spain; ggaraulet@cnio.es (G.G.); bbaez@cnio.es (B.B.B.); gmedranon@cnio.es (G.M.); 2 Protein Production Unit, Spanish National Cancer Center—CNIO, 28029 Madrid, Spain; mrivas@cnio.es (M.R.-S.); dsancheza@cnio.es (D.S.-A.)

**Keywords:** theragnosis, oncology, nuclear medicine, immunoPET, radioimmunotherapy

## Abstract

**Simple Summary:**

ImmunoPET involves tagging an antibody (a protein that targets cancer cells) with a radioactive substance that can be seen by imaging. This could non-invasively visualize where cancer is and how much of the target antigen is present, helping decide which patients should get radioimmunotherapy. This therapy uses the same antibody but with a different radioactive substance designed to kill cancer cells. ImmunoPET shows where the cancer is and how much there is, helping choose the right patients for treatment. It helps monitor how well the treatment is working, so adjustments can be made if necessary. It provides important data on how the antibody moves and clears in the body, which helps in calculating the right dose and optimizing the treatment plan. This strategy can lead to more targeted and personalized therapies with fewer side effects because it leverages the precision of antibodies and the powerful effects of radiation.

**Abstract:**

The combination of immunoPET—where an antibody (Ab) is labeled with an isotope for PET imaging—and radioimmunotherapy (RIT), using the same antibody with a therapeutic isotope, offers significant advantages in cancer management. ImmunoPET allows non-invasive imaging of antigen expression, which aids in patient selection for subsequent radioimmunotherapy. It also facilitates the assessment of tumor response to therapy, allowing for treatment adjustments if necessary. In addition, immunoPET provides critical pharmacokinetic data, including antibody biodistribution and clearance rates, which are essential for dosimetry calculations and treatment protocol optimization. There are still challenges to overcome. Identifying appropriate target antigens that are selectively expressed on cancer cells while minimally expressed on normal tissues remains a major hurdle to reduce off-target toxicity. In addition, it is critical to optimize the pharmacokinetics of radiolabeled antibodies to maximize tumor uptake and minimize normal tissue uptake, particularly in vital organs such as the liver and kidney. This approach offers the potential for targeted and personalized cancer therapy with reduced systemic toxicity by exploiting the specificity of monoclonal antibodies and the cytotoxic effects of radiation. However, further research is needed to address remaining challenges and to optimize these technologies for clinical use.

## 1. Introduction

The potential use of a single molecular targeting agent for both diagnostic and therapeutic purposes, known as theranostic drugs, represents an opportunity to improve the management of cancer patients. In nuclear medicine, radioimmunotheranostic agents are a more specific subset of theranostics that involve the coordinated use of a “matched pair” radiopharmaceutical for diagnostic imaging and antibody-targeted therapy using β- or α-emitting isotopes [1,2]. This integrated approach is the epitome of personalized medicine. By combining a specific tumor biomarker, quantitatively imaged with high sensitivity by positron emission tomography (PET), with a theranostic β- or α-emitting radionuclide pair, linked to the same targeted molecule, it becomes possible to regulate tumor development in individual patients. Traditionally, antibodies directed against exposed antigens that are overexpressed in tumors but not in healthy tissues have been the preferred targeting molecules due to their high specificity and selectivity. However, full-length antibodies used in immunoPET imaging have several drawbacks such as slow pharmacokinetics and prolonged circulating half-life due to their high molecular weight. As a result, PET imaging is typically delayed for several days after injection, resulting in increased background radiation and degraded image quality. In addition, when coupled with a therapeutic radionuclide, the circulating activity poses a risk of potentially damaging healthy tissues such as bone marrow, kidney, and liver [3].

In today’s landscape of cutting-edge molecular targeting therapy and cancer immunotherapy, there is an urgent need to advance the clinical care of cancer patients. This requires the seamless integration of novel molecular imaging techniques and innovative radioisotope-based targeted therapies. One such pioneering approach is radioimmunotheragnosis, a fusion of immunoPET and radioimmunotherapy (RIT) techniques. In this comprehensive review, we discuss the latest studies and advancements in this revolutionary technology, which is poised to redefine the landscape of cancer management [4,5,6].

For therapy, RIT uses monoclonal antibodies (mAbs) labeled with α-particle, β-, or Auger electron (AE)-emitting radionuclides to selectively deliver radiation to cancer cells. The mAbs specifically bind to cancer cell surface-expressed antigens, delivering lethal doses of radiation directly to the tumor while sparing healthy tissue [7]. Commonly used isotopes for antibody labeling include iodine-131, yttrium-90, and lutetium-177, among others. These isotopes emit different types of radiation with different ranges and energies, allowing for tailored treatment depending on the characteristics of the tumor [6]. Compared to traditional external beam radiation therapy, RIT offers the advantage of targeted delivery, potentially reducing systemic toxicity and sparing healthy tissues. Some issues with RIT include optimizing dosimetry, managing hematologic toxicities such as myelosuppression, and addressing the development of resistance.

For imaging, the application of mAbs began with radioimmunodetection, utilizing gamma cameras and later SPECT imaging by labeling mAbs with isotopes such as ^123^I, ^111^In, or ^99^mTc. Over time, the focus of the nuclear medicine community shifted towards PET radionuclides like ^64^Cu, ^89^Zr, and ^124^I. This shift occurred because immunoPET imaging provides higher spatial resolution, more precise quantification, and often better target-to-background ratios (TBR) compared to immunoSPECT [8]. ImmunoPET combines the specificity of mAbs with the sensitivity and resolution of PET imaging. Antibodies are labeled with positron-emitting radionuclides, allowing for non-invasive imaging of antigen expression in vivo [1,9]. ImmunoPET has multiple clinical applications, including cancer diagnosis, staging, treatment response assessment, and patient stratification. It provides valuable information about tumor characteristics, such as antigen expression levels and heterogeneity, which helps to plan a personalized treatment. This technique offers high sensitivity and quantitation capacity, as well as high specificity due to the targeting capability of the antibodies. A major challenge is to achieve a good match between the isotope and the size of the antibody. The combination of immunoPET and radioimmunotherapy offers several advantages. ImmunoPET provides non-invasive imaging of antigen expression, which can help select patients most likely to benefit from radioimmunotherapy. In addition, it can be used to assess tumor response to therapy and guide treatment modifications if necessary. Furthermore, immunoPET can provide valuable pharmacokinetic data, such as antibody biodistribution and clearance rates, which can be used to calculate dosimetry and optimize treatment protocols [10].

## 2. Antibodies in ImmunoPET and RIT

As a primary function, antibodies and antibody-like molecules designed and used for oncology treatment should recognize their target antigen with high specificity and binding affinity. On the other hand, antigens should have high expression in tumors and in the surrounding microenvironment [11], but low expression in normal tissues to minimize background noise in imaging. In addition, the epitopes of the antigen recognized by the antibody must be present on the tumor cell surface to facilitate the interaction and further accessibility, although some mAbs specific for cytoplasmic domains of transmembrane proteins or entirely intracellular antigens have also been generated. For instance, capromab (7E11) is an mAb that binds to an epitope on the intracellular domain of PSMA and has been investigated as a SPECT imaging tracer for prostate cancer [12] and as a therapeutic agent when labeling with ^90^Y [13]. However, the use of this mAb as a tracer is limited to detecting dead cells and does not offer advantages in imaging compared to tracers derived from extracellular domain binders. Additionally, some studies using antibodies tagged to cell-penetrating peptides have shown potential for PET or SPECT imaging of intracellular targets such as p21 [14], p27 [15] or H2AX [16]. In this regard, there are many tumor-antigens that are under investigation as targets for various molecules designed to specifically detect them. In this review, we will discuss most of these types of antibodies, focusing on their characteristics, advantages and limitations.

**Figure 1:**
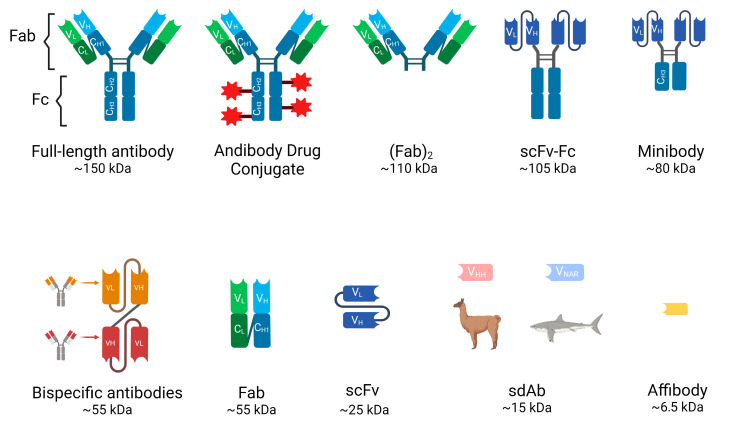
Different types of antibodies and their molecular weight (Created with BioRender.com).

Antibodies can be classified into different types based on their size, structures and mechanisms of function (Figure 1).

## 3. Full-Length Antibodies (Abs)

These glycoproteins, also known as immunoglobulins (Ig), are essential components of the immune system that recognize and neutralize pathogens such as bacteria and viruses. They have been the focus of research and medical applications for decades, including diagnostics, therapeutics and immunoassays. Different types of antibodies have unique structures, functions, and applications. In humans, antibodies are divided into five classes or isotypes: IgA, IgD, IgE, IgM and IgG, with IgG being the most common.

IgG is composed of four polypeptide chains: two heavy chains (each containing one variable domain VH and three to four constant domains CH1, CH2, CH3) and two identical light chains (each containing one variable domain VL and one constant domain CL) linked by disulfide bonds (see Figure 1). The antibody structure can also be distinguished into an antigen-binding fragment (Fab) and a crystallizable fragment (Fc), together forming a Y-shape that allows the variable region to be exposed and enables it to recognize its corresponding antigen [17]. The heterotetramer has a molecular weight of 150 kDa and a half-life in the bloodstream of approximately 21 days before being eliminated mainly by intracellular enzymatic degradation. They cannot be eliminated by the kidneys or the liver due to their large size.

Antibody binding to cancer cells can lead to cell death through various mechanisms, such as exerting neutralizing or apoptotic effects and promoting innate immune responses, including antibody-dependent cell-mediated cytotoxicity (ADCC) and antibody-dependent cellular phagocytosis (ADCP), or complement-dependent cytotoxicity (CDC). In addition, these antibodies can be engineered for various functions, such as drug conjugation, radioimmunotherapy, and immunoPET imaging.

Although these Abs have been used for most medical treatments and research, the full-length antibodies have some limitations, such as slow blood clearance and low target/background ratio (especially important to achieve good quality for PET imaging).

## 4. Antibody Fragments

Various antibody fragments can be prepared by enzymatic digestion with papain or pepsin or by genetic engineering to overcome the drawbacks of full-length antibodies. Compared to full-length Abs, they exhibit shorter serum half-lives (4–20 h), faster elimination from the bloodstream, lower immunogenicity, since most of them lack the Fc region, and the ability to homogeneously penetrate tissues, including solid tumors, allowing for better target/background ratios for imaging [1]. The most frequently used antibody fragments in clinical developments are the following (Figure 1):

Fragment Antigen-Binding (Fab and (Fab’)2): Fab is a 55-kDa monovalent fragment consisting of a complete light chain and the heavy chain variable (VH) domain and the first constant (CH1) region. It can be produced by papain digestion of an intact antibody or recombinantly in several high-yield expression systems. This fragment retains antigen-binding specificity, and the smaller size improves tissue penetration and reduces non-specific binding. Useful for certain diagnostic and therapeutic applications, although lower molecular stability may be a limitation compared to full-length antibodies. On the other hand, (Fab’)2 is another antibody derivative produced by pepsin digestion of IgG. It retains the bivalent binding capacity of IgG immunoglobulins but lacks the constant region (Fc) and is consequently smaller in size (~110 kDa).

Single-chain variable fragments (scFvs): These fragments are engineered antibodies consisting of the variable regions of the heavy and light chains connected by a variable peptide linker to form a single-chain molecule. With a molecular weight of only 25–30 kDa, scFvs have even better tissue penetration and rapid clearance from the bloodstream through the urinary system. They can be produced in bacterial systems, which is cost-effective, but their lower stability and functional affinity (avidity) compared to full-length antibodies and rapid renal clearance have limited their use in the clinic. Multivalent scFvs, constructed by connecting VH and VL fragments with short “GGGGS” peptide linkers, include diabodies (~60 kDa), triabodies (~90 kDa) and tetrabodies (~110 kDa). These can serve as advantageous vectors for radioimmunoimaging because their increased size enhances affinity and prolongs blood circulation [18,19].

Minibodies: These constructions are also engineered Ab fragments. They are produced by combining scFv molecules with human IgG1 constant heavy chain-3 (CH3). These fragments have accelerated blood clearance compared to full-sized antibodies, making them particularly useful for in vivo imaging as accumulation in the kidney can be avoided [19].

Bispecific Antibodies: Unlike monospecific antibodies, bispecific antibodies have two different antigen-binding sites, allowing them to bind to two different antigens simultaneously. The specific antigens/epitopes can be localized either on the same cell or on different cells. They are emerging as potent therapeutic agents for cancer immunotherapy. The disadvantages are complex engineering and consistent production to overcome the molecules’ instability. The bispecific antibodies targeting two different cells are mostly T cell engagers (BiTE, Bispecific T cell engagers) that link a cancer cell to an effector T cell. A cancer-specific scFv is joined with a T cell-binding scFv (anti-CD3) via a glycine-serine peptide linker [20,21]. Upon crosslinking, the T cell is activated to kill the bound target cancer cell by secreting perforin and other granzymes. These cytolytic proteins can form pores on the cancer cell membrane, resulting in cancer cell lysis. The BiTE format has emerged as a potent antibody-based therapeutic since the regulatory approval of blinatumomab (anti-CD19xanti-CD3) in B-cell malignancies [22].

**Figure 2:**
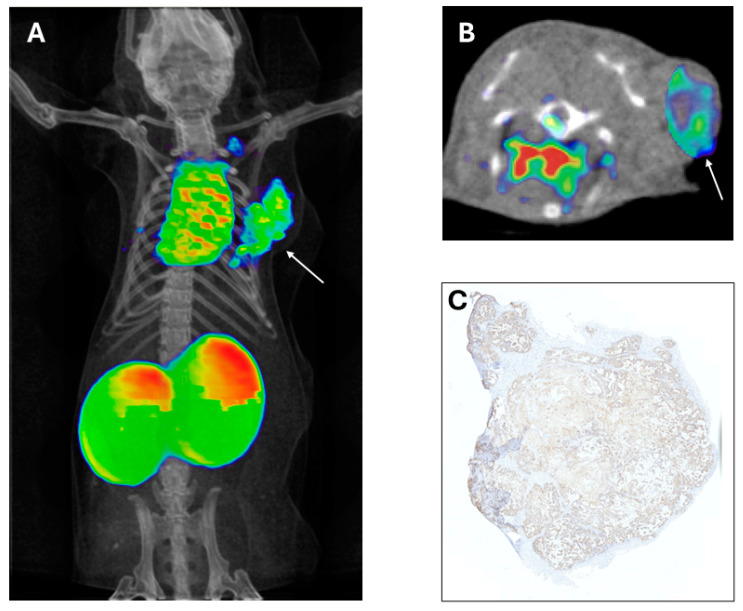
Coronal projection of ^68^Ga NOTA-3CMP75 nanobody imaging in a Triple Negative Breast Cancer tumor model (**A**). Axial view showing the high and specific probe uptake (**B**). Tumor MT1 MMP immunohistochemistry denoting extensive target expression (**C**). Arrows indicate the xenografted tumor.

Single domain antibodies (sdAb): Heavy chain antibodies are a class of antibodies found in camelids and sharks that are characterized by the absence of the light chain and the CH1 domain. The variable domain of their heavy chain can be efficiently cloned, expressed and purified in a heterologous system such as *E. coli* to yield a single domain antibody of approximately 15 kDa, also known as VHH from camelids, commercially called nanobody (Nb), and VNAR, natural heavy-chain only antibody derived from sharks. These sdAbs have unique biochemical and biophysical properties such as their stability, high solubility, thermal and proteolytic resistance [23]. Single domain antibodies are the smallest but still functional natural antibody-derived fragments that retain high specificity and good affinity binding properties, also exhibiting faster pharmacokinetics than the original structure. Their small molecular weight allows for much deeper tissue penetration, more homogeneous distribution within the tumor microenvironment, and a faster blood clearance through the renal excretion route, increasing the tumor-to-background ratio and image quality in PET experiments. In addition, nanobodies may be superior for targeting brain tumors or brain metastases from systemic tumors due to their ability to cross the brain-blood-barrier, attributed to their smaller size and different physicochemical properties compared to conventional antibodies [24]. Moreover, their shorter half-life can be matched with rapidly decaying radionuclides for diagnosis and tumor treatment [21,25,26] (Figure 2). The problem of rapid renal clearance, especially if they are developed as therapeutic tools, can be solved by PEGylation or fusion with albumin-binding proteins [27]. Another favorable feature for clinical use is its low immunogenicity, since VHH structure is highly similar to human VH structure and the small size of VHH implies a low number of potentially immunogenic epitopes [28]. Nevertheless, humanization of VHH is possible to further reduce its immunogenicity [29].

Affibody molecules (AB): they are even smaller engineered proteins (6.5 kDa) that bind with high affinity to a variety of target molecules, mimicking monoclonal antibodies [30]. Affibodies can be used for protein purification, enzyme inhibition, diagnostic imaging and ultimately targeted therapy. The robust structure of affibody molecules, composed of alpha helices and lacking disulfide bridges, allows them to be conjugated to radionuclides without compromising their binding capacity. Their small size also helps achieve high-contrast images [31,32]. For example, affibodies “ABY-002 and ABY-025” have been developed for HER2 imaging using SPECT (radiolabeled with ^111^In) and for PET (radiolabeled with ^68^Ga) [33,34].

Antibody–drug conjugates (ADCs): They consist of a small molecule drug (payload) covalently attached to a monoclonal antibody via a chemical linker to target tumor-expressing antigens and destroy cancer cells. The toxic payloads most commonly used in approved and clinical-stage ADCs are microtubule disruptors or DNA-damaging agents [35]. PET imaging of radiolabeled ADCs has been used to determine the pharmacokinetics, biodistribution and tissue uptakes of ADCs [36] as radioisotopes such as ^64^Cu, ^86^Y, ^89^Zr, and ^124^I can be conjugated to ADC without affecting the drug properties [37].

## 5. Isotopes

There are a number of isotopes used in nuclear medicine as theranostic agents. The choice of radioactive isotopes is critical for the efficacy and safety of immunoPET and RIT. Here is an overview of some of the most commonly used radioactive isotopes, focusing on their properties, advantages, and limitations (Table 1):

## 6. Commonly Used Radioactive Isotopes in ImmunoPET

- ^89^Zr (Zirconium-89)

The relatively long half-life of ^89^Zr (half-life: 78.4 h) matches well with the pharmacokinetics of intact antibodies, allowing for sufficient time for the antibody to target the tumor and for imaging to be performed at later time points. This is particularly useful for imaging slow-accumulating targets. Importantly, the long half-life also means higher radiation dose to the patient. In addition, ^89^Zr has a high energy positron emission, β+ (23%; 396 keV) and β+ (6%; 824 keV). ^89^Zr-immuno-PET tracers have been used clinically to image HER2 [38,39] receptors and VEGF-A [40]. In addition, ^89^Zr PD-1/PD-L1 has been used in different clinical trials [39,41].

- ^64^Cu (Copper-64)

The intermediate half-life of ^64^Cu (half-life: 12.7 h) is suitable for imaging with smaller antibody fragments, such as Fab fragments and single-chain variable fragments (scFv). It provides a balance between adequate imaging time and lower radiation dose. However, the shorter half-life compared to ^89^Zr may not be ideal for imaging with full antibodies. ^64^Cu has been evaluated in preclinical studies labeling full Abs as durvalumab [42] and (Fab’)_2_ for ^64^Cu-labeled CD4+ T cell targeting (38) and also in patients for trastuzumab PET imaging [43].

- ^68^Ga (Gallium-68)

The short half-life (68 min) is ideal for imaging with very small antibody fragments, peptides, or nanobodies. ^68^Ga is readily available from a ^68^Ge/^68^Ga generator, making it convenient for clinical use. It has been used extensively in preclinical studies and the HER2-nanobody has been used successfully in patients with breast cancer. The short imaging window limits the flexibility of imaging schedules and may not be suitable for intact antibodies or other large biomolecules that require more time to accumulate at the target site [44,45].

- ^124^I (Iodine-124)

The long half-life (100.3 h) is excellent for imaging with full antibodies and allows delayed imaging, which can improve contrast by allowing non-specific background signals to clear. Antibody radiolabeling with ^124^I for immuno-PET imaging has successfully produced radiotracers for tumor detection in colorectal cancer [46], thyroid cancer [47] and gastric cancer [48]. ^124^I has high energy positron emissions β+ (12%; 687 keV) and β+ (11%; 975 keV), which may result in poorer image resolution. There is also a risk of dehalogenation, where iodine is released from the antibody and can accumulate in non-target tissues, such as the thyroid.

- ^86^Y (Yttrium-86)

With a half-life of 14.7 h, ^86^Y is suitable for medium-sized antibody fragments and provides good image quality due to its appropriate positron energy β+ (32%) decay; 394–1437 keV. However, it has less favorable properties than ^89^Zr due to the high energy γ-rays produced by the isotope. ^86^Y is less widely used and less readily available than other isotopes. It also has complex decay schemes that can complicate image quantification. This radiometal has been studied in various preclinical models for labeling F(ab’)2 fragments and mAbs [49,50].

- ^18^F (Fluorine-18)

Fluorine-18 is the most commonly used PET radionuclide due to its high positron yield, low positron energy, approximately two-hour half-life, and routine cyclotron production via proton bombardment of [^18^O]H_2_O to generate [^18^F]fluoride. However, the short half-life of around 110 min limits the types of antibody fragments suitable for labeling. Additionally, the high temperatures and nonaqueous conditions typically required for incorporating [^18^F]fluoride into organic molecules are incompatible with direct antibody labeling. To circumvent these challenges, various reactions can be utilized to indirectly radiolabel antibody fragments with 18F-prosthetic groups, thereby avoiding the harsh conditions of direct labeling. Recently, aluminum chelate complexes have emerged as an effective strategy for ^18^F-labeling of antibody fragments. The aluminum-[^18^F]fluoride ([^18^F]AlF) method, pioneered by McBride et al. [51], involves the in situ reaction of ^18^F− with AlCl_3_ to form [^18^F]AlF, which is then conjugated with a bifunctional chelator. This approach has proven to be highly efficient for radiolabeling antibody fragments [52].

## 7. Commonly Used Radioactive Isotopes in Radioimmunotherapy

- ^131^I (Iodine-131)

It was the first theranostic isotope used. ^131^I is a β and ɣ emitter that has been extensively used in RIT because of its well-established chemistry and ability to be easily conjugated to antibodies. Its beta particles provide effective cytotoxicity, and the gamma emissions allow for imaging and dosimetry. Rituximab [53] and tositumomab [54] labeled with ^131^I have been tested in patients and the latter has been approved for radiopharmaceutical therapy in the USA and EU. Gamma emissions require special handling and patient isolation to avoid radiation exposure to others. Dehalogenation may lead to accumulation of free iodine in the thyroid, requiring thyroid protection.

- ^90^Y (Yttrium-90)

The ^90^Y pure beta emitter produces high-energy particles that are highly effective at killing cancer cells. The lack of gamma emission reduces radiation exposure to medical personnel and others. On the other hand, it cannot be used for imaging for the same reason. The high-energy beta particles can damage surrounding healthy tissue if the tumor is small or near sensitive structures. ^90^Y-ibritumomab tiuxetan has been used as a first-line treatment for follicular lymphoma [55].

- ^177^Lu (Lutetium-177)

^177^Lu has favorable radiation properties, with beta particles for therapeutic effects and gamma emissions for imaging and dosimetry. Its intermediate beta energy and relatively short path length make it suitable for treating smaller tumors and minimizing damage to surrounding tissues. It has been successfully used in preclinical studies and several clinical trials in non-Hodgkin lymphoma, ovarian cancer, prostate cancer and neuroendocrine tumors. [^1^⁷⁷Lu]Lu-PSMA-617 is approved for resistant prostate cancer in the US since 2022 and [^1^⁷⁷Lu]Lu-DOTA-TATE is approved for gastroenteropancreatic neuroendocrine tumors since 2018 in the EU and USA [56,57,58].

- ^188^Re (Rhenium-188)

^188^Re emits both beta particles for therapy and gamma rays for imaging. It is available from a ^188^W/^188^Re generator, providing a convenient and continuous supply. Its shorter half-life (16.9 h) is advantageous for reducing long-term radiation exposure. On the other hand, this short half-life requires rapid targeting and treatment protocols. ^188^Re has been used in many clinical trials to label various compounds such as liposomes or hydroxyethylidine diphosphonate [59,60].

- ^211^At (Astatine-211)

It is an alpha emitter with a half-life of 7.2 h. Alpha particles have a high linear energy transfer (LET) and are extremely effective at killing cancer cells with minimal damage to surrounding tissue. This makes ^211^At particularly effective for targeting micrometastases and isolated tumor cells. The short half-life requires rapid targeting, and the production and handling of ^211^At can be challenging. Its alpha emissions limit the penetration depth, making it less suitable for large tumors. Several ^211^At-based radionuclide therapies are under investigation in various clinical trials [61].

- ^225^Ac (Actinium-225)

^225^Ac is another alpha emitter with a half-life of 10 days. Its median lethal dose is various orders of magnitude higher than ^213^Bi. This is due to its longer half-life and corresponding alpha emissions from its decay products. Four high-energy alpha particles are produced in each ^225^Ac to ^209^Bi decay. The main limitation for this isotope is the penetration depth, making it less suitable for large tumors. It has been tested for targeted alpha particle therapy in prostate cancer patients [62].

- ^213^Bi (Bismuth 213)

Another emerging alpha emitter isotope with a half-life of 45 min. It can be produced using a ^225^Ac/^213^Bi generator. The short half-life requires rapid targeting. Production and handling of ^211^At can be challenging. The anti-leukemic effects of lintuzumab labeled with ^225^Ac and ^213^Bi have been studied in patients with acute myeloid leukemia [63].

## 8. Factors Influencing the Choice of Isotope

Several factors must be considered when selecting isotopes for targeted molecular imaging and therapy to optimize efficacy and safety. The half-life of the isotope should match the biological half-life of the targeting molecule to maximize tumor localization while minimizing off-target radiation exposure. Full antibodies generally require isotopes with longer half-lives, while smaller fragments or peptides can use isotopes with shorter half-lives. In imaging, lower positron energy is preferred because it improves image resolution; high-energy positrons travel further before annihilation, reducing spatial resolution. The type of radiation emitted by the isotope also plays a critical role in therapeutic efficacy and potential collateral damage; alpha particles are highly effective but have limited penetration, while beta particles can treat larger volumes but may affect surrounding tissues. In addition, the availability and production of isotopes such as ^68^Ga or ^188^Re, which can be obtained from generators, make them convenient for clinical use. Finally, dosimetry and safety considerations are paramount; radiation dose to the patient should be minimized to ensure effective imaging, and it should be noted that isotopes with longer half-lives typically deliver higher radiation doses [9].

Therefore, the choice of radioactive isotope for immunoPET depends on a balance of factors including the half-life of the isotope, the type of targeting molecule, the required image resolution, and the clinical context. ^89^Zr and ^124^I are preferred for full antibodies due to their long half-lives, while ^64^Cu and ^68^Ga are suitable for smaller fragments and peptides due to their shorter half-lives and rapid imaging capabilities [6]. Understanding these properties helps to optimize immunoPET for accurate and effective molecular imaging. The selection of radioactive isotopes for radioimmunotherapy depends on balancing therapeutic efficacy, safety, and practical considerations. ^131^I and ^90^Y are well-established in clinical practice, with distinct advantages for different tumor types and sizes. ^177^Lu offers a versatile option with both therapeutic and imaging capabilities. Emerging isotopes such as ^213^Bi, ^225^Ac and ^211^At offer promising alternatives for specific clinical scenarios, particularly for targeting micrometastases. However, another important aspect to consider is that upon the emission of an α-particle, the daughter nuclide experiences a recoil energy significant enough to break any chemical bond formed by the chelating chemistry. These ‘free’ daughter nuclides are no longer targeted to the tumor and can damage surrounding tissue [64]. Understanding the characteristics and clinical implications of each isotope helps to tailor radioimmunotherapy to achieve optimal patient outcomes [43].

## 9. Pretargeted ImmunoPET and Radioimmunotherapy (PRIT): General Strategy

Pretargeting methodologies hold promise for enhancing antibody binding to tumor cells. A key approach involves functionalizing antibodies or their fragments with biotin, avidin, or specific oligonucleotides such as phosphorodiamidate morpholinos or peptide nucleic acids. These molecules are engineered to be bispecific. The modified monoclonal antibodies are then typically administered intravenously. After a period of 24–72 h to allow for the clearance of unbound conjugates, radionuclides tagged with a complementary counterpart that specifically recognizes the conjugated mAbs are administered intravenously, intraperitoneally, or locally [36]. The small size of the radioligand ensures rapid biodistribution and clearance, typically within a few hours, thereby minimizing off-target irradiation of healthy tissues.

Unlike traditional radioimmunoconjugates, pretargeted approaches decouple the steps of tumor-targeting and payload delivery, enhancing tumor uptake while minimizing exposure to normal tissues. Critical considerations include antibody immunogenicity and specificity, radioisotope availability, and clinical feasibility. Each step can be optimized independently, making pretargeting systems adaptable to different tumor targets, types, and radioisotopes. However, despite its versatility, pretargeting presents complexities and unique challenges for clinical translation and optimal patient use [48].

## 10. Radioimmunotheragnosis Applications in Cancer

Radioimmunotheranostics has its roots in the 1930s, with the pioneering work of Hertz et al. [65]. Realizing the current potential of radioimmunotheranostics would justify the significant investments in research and development, leading to the clinical translation of new agents and improved patient outcomes. The most substantial progress is anticipated in cancers with the highest incidence and mortality rates, such as breast cancer. Furthermore, notable advancements are expected in cancers with lower incidence but very high mortality rates, including pancreatic, ovarian, small-cell lung, and hepatobiliary cancers. However, expanding the application of radiotheranostics involves numerous challenges [2,54].

We will review some of the most common applications in preclinical and clinical cancer research (Table 2).

## 11. Hematological Malignancies

Researchers have long investigated two different approaches using radiolabeled mouse monoclonal antibodies that target the CD20 antigen on B cells. In 2002, the FDA approved ^90^Y-ibritumomab tiuxetan, marking a breakthrough treatment for patients with B-cell non-Hodgkin lymphomas (NHL) [66]. In 2003, another anti-CD20-binding antibody, ^131^I-tositumomab, also received FDA approval [54,67]. Despite their excellent clinical performance and limited toxicity in several trials combining conventional radioimmunotherapy with high-dose myeloablative conditioning chemotherapy, both drugs faced commercial failure, leading to the discontinuation of ^131^I-tositumomab in 2014.

This first-in-class monoclonal antibody was a commercial failure despite no significant toxicity. This failure was attributed in part to occasional communication gaps between oncologists and nuclear medicine physicians, as nuclear medicine was often viewed primarily as a diagnostic discipline with limited therapeutic applications, with a few exceptions. This market rejection has somewhat stalled the research and development of new antibodies for radioimmunotheragnosis [3].

To address the issues of limited tumor penetration and suboptimal pharmacokinetics associated with full-length antibodies, researchers have developed a range of hCD20-targeting single-domain antibodies (sdAbs). One such sdAbs was radiolabeled with ^68^Ga for immunoPET imaging and with ^177^Lu for targeted therapy of lymphoma [68].

Building on the success of imaging, extensive preclinical evidence has demonstrated that CD38-targeted RIT can effectively eradicate disseminated multiple myeloma (MM) [69,70,71]. A novel two-step pretargeted RIT (pRIT) strategy, using a less immunogenic bispecific protein (028-Fc-C825) targeting both the CD38 antigen and the ^90^Y-DOTA ligand, along with ^90^Y-DOTA-biotin, significantly reduced tumor growth and prolonged survival in both MM and non-Hodgkin lymphoma (NHL) models [69]. These findings highlight the superiority of CD38 for both immunoPET imaging and pRIT. The clinical translation of these CD38-targeted theranostic agents promises to improve the management of patients with MM (NCT03665155) [70,72,73].

## 12. Breast Cancer

Over the past two decades, the human epidermal growth factor receptor 2 (HER2/ErbB2) has emerged as a key target for molecular imaging. Alongside the clinical approval of HER2-targeted antibody therapeutics such as trastuzumab, trastuzumab emtansine (T-DM1), and pertuzumab, numerous antibody-based radiotracers have been developed to visualize HER2 expression [74]. Notably, two HER2-specific VHHs, 2Rs15d and 5F7, have been radiolabeled with [^18^F]TFPFN for immunoPET imaging, demonstrating effective tumor identification with reduced renal uptake. Additionally, [^18^F]AlF-NOTA-Tz-TCO-GK-2Rs15d has been validated as a suitable vehicle for radioimmunotherapy (RIT) when labeled with ^177^Lu or ^131^I [57,68,75]. These developments provide valuable therapeutic options to complement immunoPET imaging. Several clinical trials are underway using RIT in breast cancer, including a study evaluating the safety and distribution of ^131^I-SGMIB-2Rs15d, which has completed patient enrollment (NCT02683083). pRIT is also being explored for breast cancer; a recent study reported that pRIT with anti-HER2-DOTA-pRIT + ^177^Lu-DOTA-Bn inhibited HER-2 positive breast cancer and significantly improved survival without inducing toxicity in normal tissues [48]. Our group has also contributed to the development of a theranostic pair for immunoPET and RIT in a triple-negative breast cancer (TNBC) mouse model targeting MT1-MMP metalloprotease with promising results using three doses of [^177^Lu]Lu-DOTA-LEM2/15 [76].

In the phase II segment, pre/peri-menopausal women and eligible men will receive [^177^Lu]Lu-NeoB in combination with capecitabine and, for women, a gonadotropin-releasing hormone agonist (GnRHa) according to local clinical protocols. The trial aims to evaluate the efficacy of this combination therapy in adult patients with ER+/HER2−, gastrin-releasing peptide receptor-positive (GRPR+) metastatic breast cancer (mBC) following progression on CDK4/6 inhibitor-based treatment. The study will explore the preliminary efficacy of two different dose levels and administration regimens [77,78].

The treatment regimen includes [^177^Lu]Lu-NeoB and capecitabine, with [^68^Ga]Ga-NeoB used as the imaging agent. Participants will receive [^177^Lu]Lu-NeoB along with capecitabine, and GnRHa will be administered according to local practice guidelines for eligible phase II women and men. [^68^Ga]Ga-NeoB is being investigated as a PET imaging agent to select patients for [^177^Lu]Lu-NeoB therapy, particularly those with GRPR-overexpressing tumors, including mBC. Preclinical and clinical studies have demonstrated the favorable technical and diagnostic performance of [^68^Ga]Ga-NeoB, producing high-quality images that allow for accurate interpretation NCT06247995 [79,80].

## 13. Prostate Cancer

Several mAbs have been developed to target both intracellular and extracellular epitopes of Prostate-Specific Membrane Antigen (PSMA) [81,82,83]. Among these, J591, a humanized mAb, has shown promise in clinical investigations for both imaging and therapeutic applications. Studies by Fung et al. [84] have demonstrated comparable surface binding and internalization rates for radiolabeled variants of J591, supporting the feasibility and safety of using ^177^Lu- and ^124^I- or ^89^Zr-labeled J591 for theranostic purposes. This approach potentially offers improved targeting of bone lesions compared to traditional imaging methods NCT00538668. These advances could revolutionize management strategies for patients with PSMA-positive prostate cancer. Conversely, capromab, another mAb, has been investigated for its utility as a SPECT imaging and therapeutic agent when labeled with ^90^Y. However, its binding to an intracellular domain of PSMA limits its efficacy in detecting soft-tissue and bone metastases compared to mAbs targeting extracellular domains since tracers derived from this mAb are limited to detecting dead cells [12,85].

Currently, a phase II trial is underway to evaluate the therapeutic efficacy of ^177^Lu-TLX591, a radiolabeled PSMA-targeting antibody, in combination with external beam radiation therapy (EBRT) in patients with biochemically recurrent, oligometastatic, PSMA-expressing prostate cancer. TLX591 represents a potential breakthrough in the treatment of PSMA-expressing tumors NCT05146973.

Even though there is no antibody, the strategy followed for theragnosis in prostate cancer with PSMA could be suitable for the prostate cancer antibodies, moving from ^177^Lu to an alpha emitter. The dosimetry of ^213^Bi-PSMA-617 is within the range typically considered appropriate for clinical use. However, compared to ^225^Ac-PSMA-617, it presents challenges such as increased off-target radiation influenced by perfusion and a prolonged biological half-life of PSMA-617 in organs where dose limits are critical, exceeding the physical half-life of ^213^Bi. As a result, while still viable, ^213^Bi is emerging as a secondary option for radiolabeling in targeted alpha therapy for prostate cancer [86].

## 14. Hepatocellular Carcinoma

There are several clinical trials involving anti-CEA therapies and other radiolabeled antibody fragments for hepatocellular carcinoma (HCC): Anti-CEA CIGB-M3 ScFv17II-131CRC (2011): This was a phase I clinical trial of the ^131^I-labeled anticarcinoembryonic antigen CIGB-M3 multivalent antibody fragment [87]. A specific trial was performed to probe the clinical value of iodine [^131^I] metuximab infusion combined with transcatheter arterial chemoembolization (TACE) for patients with post-intervention relapse of mid- or advanced-stage hepatocellular carcinoma. Adjuvant ^131^I-Metuximab for hepatocellular carcinoma after liver resection (2020, NCT00819650): this was a randomized, controlled, multicenter, open-label, phase II trial assessing the efficacy of adjuvant ^131^I-metuximab for hepatocellular carcinoma following liver resection (2020) NCT00819650 [88,89].

## 15. Colorectal Cancer

In clinical trials involving RIT for colorectal cancer, one notable study investigated the use of radiolabeled scFv. Conducted in 2011, this phase I trial assessed ^131^I-CIGB-M3, a trivalent scFv specific for CEA, in patients with colorectal metastases [87]. The feasibility study involved 17 patients divided into two groups, each receiving either 0.3 mg or 1 mg of CIGB-M3 scFv, with similar injected activities of about 185–259 MBq of ^131^I. Toxicity assessments indicated low off-target toxicity for the scFv fragment in both groups and showed lower human anti-mouse antibody (HAMA) responses compared to those receiving a single 1 mg dose of the parental CB-CEA-1 full antibody. Despite the promising pharmacokinetic results and favorable dosimetry (0.07 ± 0.02 to 0.08 ± 0.02 mGy/MBq in the whole body, 1.1 ± 0.6 to 2.0 ± 1.3 mGy/MBq in the kidneys), further clinical development of the trivalent CIGB-M3 scFv did not proceed.

Subsequently, a new generation of bispecific Ab (bsAbs) was developed to reduce the immunogenicity observed with the chimeric F(ab′)_2_. The trispecific humanized TF2 Fab′ comprises an anti-histamine-succinyl-glutamine (HSG) fragment derived from the 679 anti-HSG IgG_1_,along with two fragments of the humanized anti-CEA derived from hMN-14 IgG_1_ (labetuzumab) [90]. A first-in-human study by Schoffelen et al. (NCT00860860), published in 2013, demonstrated the feasibility and safety of pRIT using TF2 and ^177^Lu-IMP-288 (a 1.5 kDa HSG peptide) in patients with metastatic CRC [91]. An earlier imaging study with TF2/^111^In-IMP-288 identified a 24-h interval between the two treatments as most suitable. Patients received a mean injected activity of 5.6 GBq with no significant differences observed between cohorts. However, the HAMA response to TF2 was notably high 1 week post-administration and continued to increase over the 8-week follow-up period, indicating unexpected immunogenicity despite the humanized nature and lack of an Fc portion in TF2.

## 16. Lung Cancer

Investigators have demonstrated that pRIT using a bispecific antibody can deliver a higher radiation dose to tumors compared to a directly radiolabeled anti-CEA antibody, resulting in improved anti-tumor efficacy. A clinical trial (NCT01221675) is designed to evaluate pRIT using a novel recombinant anti-CEA bsAb and a ^177^Lu-labeled peptide for treating CEA-expressing small cell lung cancer (SCLC) and non-small cell lung carcinoma (NSCLC).

The L19SIP antibody, a fully human antibody, selectively targets tumor blood vessels while sparing normal tissue. Since neovascularization is rare in adults, except in the female reproductive tract, but common in aggressive cancers, this antibody has significant therapeutic potential. A feasibility study (NCT01124812) aims to evaluate the efficacy of ^131^I-labeled L19SIP in combination with radiochemotherapy for patients with newly diagnosed, unresectable, locally-advanced NSCLC, building on promising results from previous studies.

A phase I trial (NCT00738452) of RIT using Y90-Mx-DTPA-cT84.66 is underway for patients with unresectable or medically inoperable, non-metastatic, CEA-producing stage I-IIIB NSCLC. This trial follows the completion of radiotherapy alone or combined with systemic therapy.

Finally, a recent clinical trial in 2024 (NCT05130255) involves treating patients with SCLC, sarcoma and malignant melanoma using the GD2-SADA:^177^Lu-DOTA complex. This two-step radioimmunotherapy, administered as two separate products GD2-SADA and ^177^Lu-DOTA, aims to assess safety and tolerability.

## 17. Others

Adults with leptomeningeal metastases from solid tumors are treated with 177Lu-DTPA-omburtamab, a radioactive labeling of a murine monoclonal antibody targeting B7-H3. Breast, NSCLC, malignant melanoma NCT04315246.

**Figure 3:**
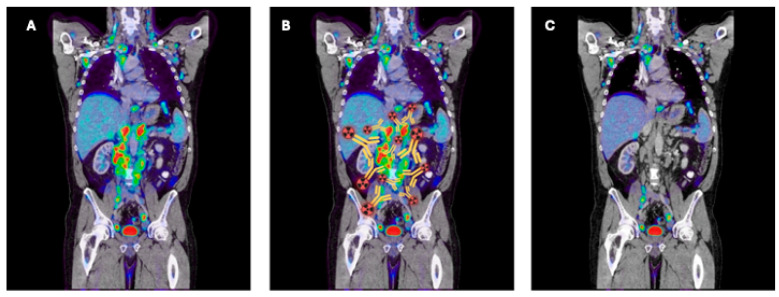
Example of (**A**) immunoPET imaging before and (**C**) after RIT with the same compound showing the decrease of activity after therapy in metastatic lymph nodes. (**B**) shows the way the therapeutic agent finds the antigen. (Created with BioRender.com).

In (Figure 3) we show a figurative example of how an immunoPET image visualizes the target, in this case the para-aortic lymph nodes, and how they disappear after RIT.

## 18. Dosimetry

Radionuclide therapies deliver cytotoxic radiation to target tissues while minimizing damage to healthy tissues. The dose of radiation absorbed by tissue, measured in grays (Gy), is critical for determining the biological effects of radiation, such as cell death. Therefore, accurate dosimetry, or the estimation of absorbed radiation, is essential for optimizing the safety and effectiveness of radiation-based treatments.

In external-beam radiation therapy, dosimetry is a well-established part of routine treatment planning. Parameters like radiation duration, intensity, the region to be irradiated, and the target volume are relatively straightforward to manage. However, radionuclide treatments are more complex due to the physical and pharmacokinetic interactions of radiopharmaceuticals throughout the body. This complexity makes it challenging to translate dosimetric measurements from research to routine clinical practice, where treatment planning often relies on the total administered radiation (activity) rather than site-specific absorbed doses [92].

Traditionally, dosimetric techniques in nuclear medicine have focused on determining the maximum tolerated doses of radionuclides for individuals. Numerous studies have evaluated the pharmacokinetic properties and biodistribution of both classic and newly developed agents. While this approach is prevalent in research, it has led to the development of simpler, widely adopted clinical activity-based protocols. These protocols estimate the highest activities that can be safely administered to a patient without exceeding radiation limits for critical organs, such as approximately 40 Gy to the kidneys (in patients without risk factors for renal toxicity) and 2 Gy to the bone marrow. ImmunoPET imaging facilitates dosimetry calculations for these therapies and predicts dose-limiting organs before RIT [93], optimizing the development and use of RIT agents.

Advances in imaging technology, particularly hybrid imaging, along with improvements in dosimetry software and new diagnostic radiopharmaceuticals, have enhanced the definition of target volumes and their integration with pharmacokinetic and irradiation data. For internal dosimetry to play a central role in routine theranostic planning, further studies and some technical simplifications are needed [69,94].

## 19. Future

The production, distribution, and storage of radiotheranostic agents encounter several challenges. Significant disparities exist within and between countries in terms of the availability of medical cyclotrons, GMP-compliant production facilities, and dedicated treatment centers that adhere to radiation safety standards. These differences lead to substantial cost variations between commercial suppliers and in-house manufacturers. Reliable distribution networks are essential to ensure the safe and timely delivery of these agents, particularly as demand rapidly increases. Radiotheranostic agents have a limited shelf life due to the radioactive half-life of the radionuclides involved (Table 1). Unlike conventional cancer therapies, both manufacturing (central vs. local) and logistics (delivery, application, and waste management) must be tailored to accommodate these short shelf lives, which restrict the number of patients treated per production cycle [3]. Additional challenges include the necessity for nuclear medicine physicians who are thoroughly trained in therapy planning, dosimetry, and response assessment, as well as facilities that comply with national radioactive waste management regulations. Moreover, advancements in PET technology and reconstruction algorithms are improving the spatial and temporal resolution of immunoPET images [95]. Recently introduced long-axial field-of-view (LAFOV) PET/CT systems represent a significant advancement in nuclear medicine. Their higher sensitivity allows for substantial reductions in applied activity and shorter scan durations, enabling the capture of a wider dynamic range of tracers, the use of lower radiopharmaceutical doses, and improved quantification. For the first time, these systems facilitate whole-body dynamic imaging with high temporal resolution, which is particularly important for the in vivo evaluation of new radiopharmaceuticals. However, challenges such as high purchase costs, logistical issues, and optimal operation within a nuclear medicine department must be considered [96]. ImmunoPET imaging is predominantly used in patients with metastatic disease, who frequently have lesions that lack histochemical confirmation. The primary goal of initial immunoPET imaging is to stratify patients by mapping the expression of specific biomarkers. A reduction in tracer uptake or lesion size on post-treatment images can indicate that these lesions are histopathologically positive.

Designing robust antibody fragments with high affinity to antigens, favorable pharmacokinetic profiles, low toxicity, and long-term stability is challenging. These fragments must endure harsh radiochemical processes without compromising their properties. Small antibody fragments, such as scFv or single-domain antibodies (nanobodies), are particularly susceptible to reduced antigen affinity following radiochemical processing [26,97,98]. Additionally, the scarcity and heterogeneity of clinical studies make it difficult to assess the efficacy of antibody fragments for RIT. The limited number of studies, small patient populations, and the diversity of fragment formats and targets contribute to the lack of substantial evidence supporting the effectiveness of RIT with fragments. Despite these challenges, antibody fragments remain promising for RIT or nuclear imaging applications; however, optimization is needed. Recent advancements in phage-display technology, chemical and chemoenzymatic engineering, and radiolabeling protocols have addressed many of these issues, yielding promising results in preclinical studies [2,99,100].

For the development of antibody-based radiopharmaceuticals, multidisciplinary collaboration is crucial. Various approaches, including genomic, serological, proteomic, biological, and bioinformatical methods, should be utilized to identify antigens that are highly or exclusively overexpressed on the surface of the tumor cells, tumor stromal cells, tumor vascular endothelial cells, immune cells, or beta cells.

Combination with established systemic therapies is a promising strategy that could enhance patient outcomes. Current targeted radionuclide approaches being evaluated in preclinical studies and early-phase trials include combinations with chemotherapy, radiosensitizers, EBRT and immunotherapies.

Novel targets and approaches for developing radiotheranostics have primarily focused on directing α- or β-radiation emitters to the surface of tumor cells, followed by intracellular trafficking and retention, resulting in DNA damage. Emerging radiotheranostic strategies are broadening the range of targets to include elements within the tumor microenvironment, such as blood vessels, cancer-associated fibroblasts (CAFs), the stromal matrix and immune cells [26,100,101]. Targeting stromal cells in the tumor microenvironment is particularly promising since these cells are generally more genetically stable than tumor cells, which can downregulate or lose expression of certain targets. Additionally, stromal cells contribute to the development of an immunosuppressive microenvironment and to drug resistance. These novel approaches could enhance the efficacy and durability of radiotheranostic treatments [102].

## 20. Conclusions

The careful application of radioimmunotheranostics in cancer patients has the potential to significantly enhance treatment outcomes. However, several major challenges must be addressed. There is an urgent need to generate robust evidence to enable a broader range of radiotheranostic agents to gain regulatory approval and enter the market swiftly. Additionally, strategies are needed to improve the global availability of radiotheranostics. The current success of radiotheranostics is likely to attract increased interest from both academia and industry in identifying and developing novel targeted agents. This is expected to lead to earlier and more accurate cancer detection, personalized treatments, and improved patient outcomes.

## Figures and Tables

**Table 1:** List of commonly used radioisotopes for immunoPET and RIT and their energy emission and half-life.

Common Use	Radioactive Isotopes		Half-Life	Emission
ImmunoPET	^89^Zr	Zirconium-89	3.3 days	β+
	^64^Cu	Copper-64	12.7 h	β
	^68^Ga	Gallium-68	68 min	β+
	^124^I	Iodine-124	4.2 days	β+
	^86^Y	Yttrium-86	14.7 h	β+
	^18^F	Fluorine-18	110 min	β+
Radioimmunotherapy	^131^I	Iodine-131	8 days	β and ɣ
	^90^Y	Yttrium-90	2.7 days	β
	^177^Lu	Lutetium-177	6.7 days	β and ɣ
	^188^Re	Rhenium-188	16.9 h	β and ɣ
	^211^At	Astatine-211	7.2 h	α
	^225^Ac	Actinium-225	10 days	α
	^213^Bi	Bismuth-213	45 min	α

**Table 2:** Ongoing clinical trials of RIT with different radiolabeled antibodies.

Target Antigen		Tracer Name	Patient Population	Trial Phase
Carbonic anhydrase IX (CAIX)		^177^Lu-girentuximab mAb (Girentuximab)	Clear Cell Renal Cell Carcinoma	phase II
		^131^I-cG250 mAb	Kidney cancer	phase II
Carcinoembryonic antigen (CEA)		^131^I-CIGB-M3 scFv	CEA+ colorectal cancer	phase I
		^131^I-A5B7 mAb	Gastrointestinal carcinoma	phase I
		^131^I-labetuzumab mAb	Chronic myelogenous leukemia	phase II
Clusters of differentiation (CD)	CD20	^90^Y-Rituximab mAb	B-cell Non-Hodgkin lymphoma	phase I
		^90^Y-ibritumomab tiuxetan mAb	CD20+ Non-Hodgkin lymphoma and B-cell lymphoma	phase II/phase III
	CD22	^227^Th-labeled CD22-targeting antibody mAb	CD22+ Non-Hodgkin lymphoma	phase I
		^111^In/90Y-epratuzumab mAb	Non-Hodgkin lymphoma	phase II
	CD33	^225^Ac-lintuzumab mAb (Lintuzumab)	Acute myeloid leukemia	phase I
	CD37	^177^Lu-lilotomab satetraxetan mAb (Lilotomab)	Non-Hodgkin lymphoma	phase II
	CD44	^186^Re-bivatuzumab mAb	Head and neck squamous cell carcinoma	phase I
	CD45	^131^I-BC8 mAb (Apamistamab)	Acute myeloid leukemia	phase I
Epidermal growth factor receptor (EGFR) family	HER2	^212^Pb-trastuzumab mAb	Peritoneal carcinomatosis	phase I
		^89^Zr/^177^Lu-trastuzumab mAb	Breast cancer	phase I
Fibroblast activation protein (FAP)		^131^I-sibrotuzumab mAb	^131^I-sibrotuzumab mAb	phase I
		^131^I-mAbF19 mAb	^131^I-mAbF19 mAb	phase I
Prostate-specific membrane antigen (PSMA)		^111^In/^177^Lu-J591 mAb (Rosopatamab)	^111^In/^177^Lu-J591 mAb	phase II
